# Hyperthermic Intraperitoneal Chemotherapy (HIPEC) and Cytoreductive Surgery (CRS): Age-Related Outcomes and a Look into the Future

**DOI:** 10.3390/cancers17030486

**Published:** 2025-02-01

**Authors:** Salvador Aguirre, Jill K. Haley, Julie A. Broski, Jordan Baker, Luke V. Selby, Shahid Umar, Mazin F. Al-Kasspooles

**Affiliations:** 1 School of Medicine, The University of Kansas, Kansas City, KS 66160, USA; saguirre2@kumc.edu; 2 Department of Surgery, The University of Kansas Medical Center, Kansas City, KS 66103, USA; jhaley@kumc.edu (J.K.H.); jbroski@kumc.edu (J.A.B.); lselby@kumc.edu (L.V.S.); 3 Department of Biostatistics and Data Science, The University of Kansas Medical Center, Kansas City, KS 66103, USA; jbaker19@kumc.edu; 4 Departments of Surgery and Cancer Biology, The University of Kansas Medical Center, Kansas City, KS 66103, USA; sumar@kumc.edu

**Keywords:** cytoreductive surgery, hyperthermic intraperitoneal chemotherapy, peritoneal carcinomatosis, age-related outcomes, patient selection, surgical oncology, readmission risk, discharge planning, peritoneal cancer index, survival analysis

## Abstract

Introduction: Peritoneal carcinomatosis presents significant treatment challenges. Cytoreductive surgery (CRS) with hyperthermic intraperitoneal chemotherapy (HIPEC) offers a promising therapeutic approach. Patient selection remains critical, and the role of age as an exclusion criterion requires further investigation. This study evaluates whether age influences postoperative outcomes in CRS-HIPEC patients. Methods: A retrospective review of a prospective comprehensive database of 271 CRS-HIPEC procedures performed between 2018 and 2023 was conducted. Logistic regression assessed the relationship between age and postoperative outcomes. Age groups (18–44, 45–69, ≥70 years) were compared based on demographic data, primary tumor site, Peritoneal Cancer Index scores, and key outcome measures. Results: Across the different age groups, there were no significant differences in PCI scores, recurrence, disease-free survival, 30-day morbidity, or mortality. Length of stay was longer in older patients (*p* = 0.009). Patients aged ≥70 had higher readmission rates (*p* = 0.041) and were more often discharged to transitional care facilities (*p* = 0.001). Older patients were also more likely to experience Clavien–Dindo grade III or higher complications (*p* = 0.008). Logistic regression confirmed these findings. Continuous age analysis yielded similar results and revealed significant differences in race and primary organ involvement. Conclusions: Age is not a significant predictor of 30-day morbidity, mortality, or survival outcomes in patients undergoing CRS-HIPEC. However, older patients require closer attention to discharge planning and readmission risk management. This study highlights the importance of comprehensive patient assessment beyond age and underscores the need for further research to better understand factors influencing outcomes in this population.

## Simple Summary

Cytoreductive surgery (CRS) combined with hyperthermic intraperitoneal chemotherapy (HIPEC) is a promising yet invasive treatment for patients with peritoneal carcinomatosis. Given the procedure’s complexity and associated risks, careful patient selection is critical to achieving optimal outcomes. Historically, age 70 was used as a strict cutoff for eligibility, limiting access for older adults. However, modern approaches focus on evaluating additional factors, such as comorbidities, frailty, and functional status, recognizing that age alone may not determine a patient’s ability to tolerate treatment. Despite this shift, the influence of age on outcomes—such as recovery, readmission rates, and survival—remains underexplored. This study aims to investigate the role of age in CRS-HIPEC outcomes, refining patient selection criteria and guiding postoperative care strategies. The findings will inform clinical decisions and support the development of tailored treatment approaches for individuals undergoing CRS-HIPEC.

## 1. Introduction

The peritoneal membrane is a relatively common site for cancer, hosting both primary malignancies like mesotheliomas and secondary malignancies resulting from metastatic disease from many sites of cancer origin. Despite rigorous therapeutic interventions, peritoneal cancers generally have a grim prognosis. These malignancies are relatively resistant to systemic therapy with survival of usually less than six months if left untreated [1,2]. Currently, there are limited operative indications for such advanced cases [1,2].

To improve both survival rates and quality of life for patients with intra-abdominal malignancies, more assertive peritoneal therapies have been introduced. Among these treatments, cytoreductive surgery (CRS) is notable for its complexity, as it involves peritonectomy and the selective resection of affected organs to minimize residual disease [3]. CRS is commonly followed by hyperthermic intraperitoneal chemotherapy (HIPEC), a single intraoperative treatment that administers heated chemotherapy directly into the abdominal cavity, thus evading the relative blood–peritoneal barrier. This approach increases drug concentration at the peritoneal surface, effectively targeting the remaining microscopic disease following the cytoreduction [4].

Comparative studies highlight the safety advantages of CRS-HIPEC over oncologic procedures with similar risks across various safety metrics [5]. However, the success of this procedure is highly dependent on careful patient selection due to its invasiveness, the extent of resection required, the potential toxicity of chemotherapy, and the intricate nature of peritoneal anatomy [6].

Effective patient selection involves evaluating factors across the preoperative oncologic timeline. Preoperatively, clinical tools and evaluations such as imaging studies, patient frailty assessments, and comorbidity indices play a critical role in identifying suitable candidates. These assessments help predict surgical feasibility and potential postoperative recovery, ensuring that CRS-HIPEC is offered to patients most likely to benefit from the procedure while minimizing undue risks.

Intraoperatively, the Peritoneal Cancer Index (PCI) and surgical assessment of disease burden are critical for determining the feasibility of achieving complete cytoreduction, and the procedure may be aborted if extensive disease makes complete cytoreduction unattainable. Additionally, the Completeness of Cytoreduction (CC) score can guide the decision on whether HIPEC is appropriate for the patient, as it is effective only in treating microscopic diseases.

While tools like the PCI and CC scores are essential for intraoperative decision-making, they are not useful for patient selection in the preoperative stage as they rely entirely on intraoperative findings. This limitation highlights the need for alternative preoperative criteria to identify suitable candidates. Historically, patients over 70 were excluded from CRS-HIPEC due to concerns about complications and outcomes. However, advances in perioperative care and surgical techniques have improved complication management and reduced mortality, enabling many centers, including international institutions, to successfully perform CRS-HIPEC on patients over 75 with limited disease and strong clinical profiles [7]. Consequently, patient selection criteria have evolved, with frailty and comorbidities now taking precedence over age alone.

Our institution’s prospective comprehensive database, with its high volume of CRS-HIPEC cases, offers more detailed insights than national datasets. This resource allows for a nuanced analysis of how age impacts surgical outcomes. Using this dataset, our study explores whether age alone should ever serve as a significant exclusion criterion in CRS-HIPEC patient selection.

## 2. Methods

This is a retrospective cohort study of a comprehensive prospective database, which includes all 271 patients who underwent CRS-HIPEC at the University of Kansas Health System between 2 January 2018, and 28 December 2023, regardless of tumor location, pathology, or prior systemic therapy. Patient data were obtained from the institutional CRS-HIPEC database and supplemented with manual chart review. Collected variables included demographics (age, sex, race), tumor characteristics (primary organ involvement, Peritoneal Cancer Index, and tumor histology), surgical details (peritonectomy locations, visceral resections, bowel resection status, and number of anastomoses), and postoperative outcomes (complications, length of stay [LoS], discharge disposition, recurrence, disease-free and overall survival time, readmission, and mortality). Readmission was defined as any admission to a hospital, including outside hospitals, for a reason other than elective procedures that occurred within 90 days of the date of discharge. Complications were evaluated using the Clavien–Dindo (C-D) classification system occurring within 30 days of the date of surgery.

Patient confidentiality was maintained through de-identification procedures, and IRB approval was obtained with informed consent waived due to the study’s retrospective nature (IRB approval code: [STUDY00161108]).

### 2.1. Study Population

Patient selection for CRS-HIPEC at our institution involved a multidisciplinary evaluation, taking into account factors such as overall performance status, tumor burden, and expected surgical feasibility. Although specific criteria were not standardized, the decision to proceed with surgery relied on a comprehensive review of individual cases by a multidisciplinary tumor board.

### 2.2. Perioperative Management

Enhanced Recovery After Surgery (ERAS) protocols were consistently implemented at our institution during the study period. These protocols included preoperative optimization measures, intraoperative fluid management, standardized postoperative pain control, early mobilization, and nutritional support. Although specific details of prehabilitation programs were not documented for this study, the ERAS protocol inherently aimed to enhance recovery outcomes. Importantly, there were no significant changes to the perioperative management protocols during the study period, including surgical techniques, antibiotic regimens, or postoperative nutritional support.

### 2.3. Scoring Systems and Outcome Measures

Several scoring systems were used to evaluate patient outcomes and surgical feasibility. The Peritoneal Cancer Index was used to assess the extent of peritoneal disease. The PCI categorizes the abdomen and pelvis into 13 regions and assigns scores intraoperatively based on the size and distribution of tumor nodules (see Appendix A). In this study, PCI volumes were categorized as low (0–9), moderate (10–19), and high (≥20), with higher scores indicating more extensive disease and a potentially lower likelihood of achieving complete cytoreduction [6].

The Completeness of Cytoreduction score ranges from 0 to 3, with CC-0 indicating no visible tumor, CC-1 indicating minimal residual tumor (less than 2.5 mm), CC-2 indicating moderate disease (between 2.5 mm and 2.5 cm), and CC-3 indicating that tumors larger than 2.5 cm remain (see Appendix B) [8]. Achieving a CC-0 or CC-1 score was critical for the success of HIPEC in this study.

Postoperative complications were classified using the Clavien–Dindo classification system, which categorizes complications from grades I to V, with higher grades reflecting increased intervention and risk (see Appendix C). While PCI and CC scores were integral in assessing intraoperative outcomes and feasibility, the C-D classification was used to evaluate postoperative outcomes and guide risk stratification [9].

### 2.4. HIPEC Parameters

The HIPEC procedure was performed following cytoreductive surgery in all patients using a closed-circuit continuous perfusion system. This system included inflow and outflow tubes, temperature probes, a heat exchanger, and a roller pump to maintain controlled circulation and consistent drug delivery. The perfusion was conducted at a target temperature of 42 °C to optimize the cytotoxic effects of chemotherapy.

The chemotherapy agents used varied based on clinical considerations, reflecting the individualized nature of treatment. Agents included Mitomycin-C (*n* = 133), Cisplatin (*n* = 40), Melphalan (*n* = 46), and Oxaliplatin (*n* = 1). In some cases, Mitomycin-C and Cisplatin were combined (*n* = 11). Specific dosing regimens and administration methods differed across patients and were tailored to individual needs and tumor biology. For example, Mitomycin-C was typically delivered with an initial dose followed by a maintenance dose at a specified interval. Similarly, Cisplatin, Melphalan, and Oxaliplatin were administered in various volumes and concentrations of a 1.5% dextrose dialysis solution, with infusion durations ranging from 60 min for Melphalan to 90 min for Mitomycin-C, Cisplatin, and Oxaliplatin.

At the conclusion of the perfusion, 2 L of normal saline were used for irrigation to minimize residual contamination and wash out the chemotherapy agents. The abdomen was then reopened for a final irrigation with 2 L of warm water or normal saline, depending on the clinical scenario. Following this, the abdomen was carefully closed in layers to ensure proper healing and minimize the risk of complications.

### 2.5. Statistical Analysis

Categorical variables, such as complications and discharge disposition, were analyzed using Pearson’s Chi-squared test or Fisher’s exact test. Continuous variables, such as PCI scores and LoS, were assessed using the Kruskal–Wallis rank sum test or linear regression. Logistic regression was employed to evaluate the association between age and categorical outcomes, with results presented as odds ratios (ORs) and 95% confidence intervals (CIs). For survival analysis, Cox proportional hazard models were used to report hazard ratios (HRs) and 95% CIs.

Given the variability in survival outcomes based on tumor type, stratified analyses were performed to assess these differences across age groups (18–44, 45–69, and ≥70 years). *p*-values were reported to confirm comparability of tumor distribution across these groups.

## 3. Results

The key patient and tumor characteristics, and findings, are summarized in Table 1, Table 2, Table 3, Table 4, Table 5, Table 6 and Table 7.

### 3.1. Patient Demographics and Primary Tumor Characteristics

**Figure 1:**
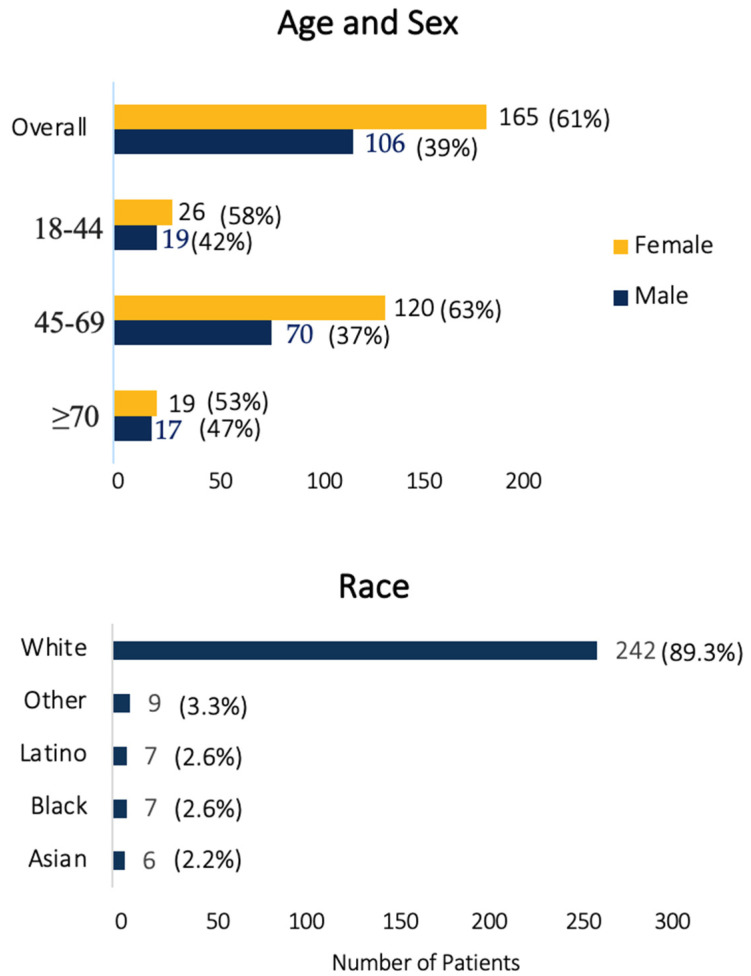
Patient demographic information.

The cohort comprised 271 patients, divided into three age groups: 18–44 years (*n* = 45), 45–69 years (*n* = 190), and ≥70 years (*n* = 36). The majority of participants were female (61%) and white (89%). Additional demographic information is provided in Figure 1.

**Figure 2:**
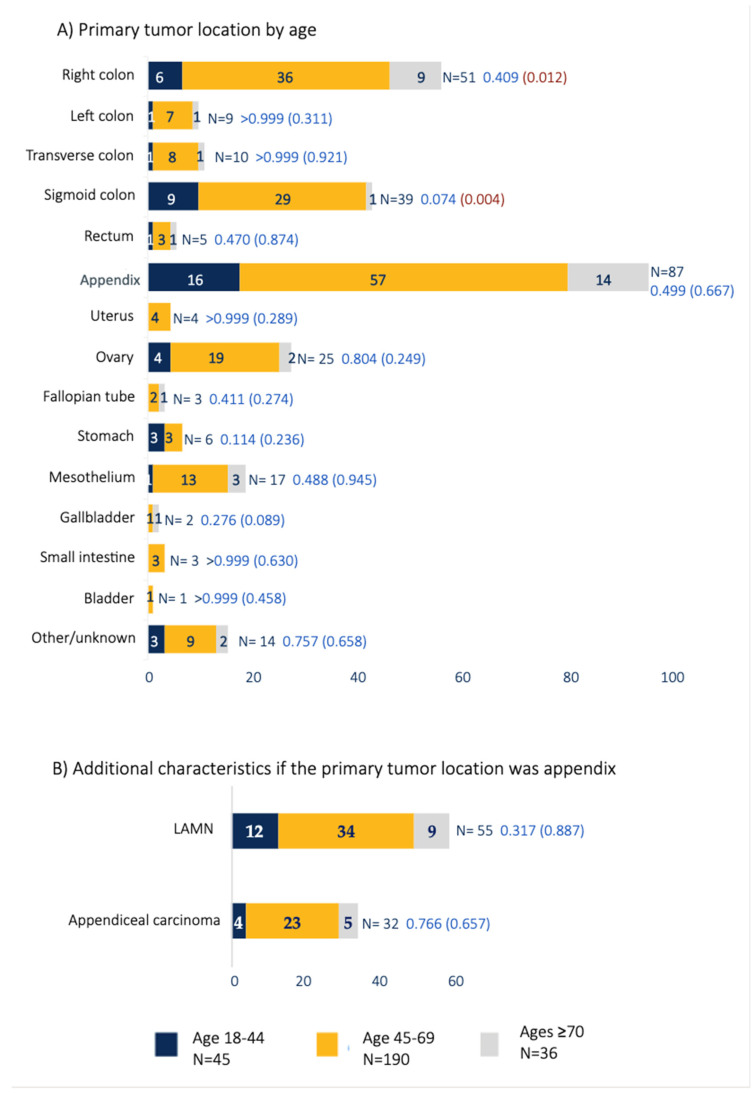
(**A**) Primary tumor locations categorized by age group (18–44, 45–69, ≥70). (**B**) Tumor characteristics for patients with primary appendix tumors.

Primary tumors (Figure 2A) were identified across various sites, with the appendix (32%) and right colon (19%) being the most common. Among those with appendiceal tumors, 63.2% were classified as low-grade appendiceal mucinous neoplasms (LAMNs) and 36.8% as appendiceal carcinoma (Figure 2B). For colorectal cancers, tumor location varied across the right, left, transverse, sigmoid, and rectal segments.

### 3.2. Peritoneal Cancer Index and Surgical Complexity

The PCI had a mean score of 11.3 (SD 7.9) across the cohort, with no significant difference between age groups (*p* = 0.544) (Table 1). The median PCI score was 10.0 (IQR: 5.0, 16.0), with similar distributions across age groups: 9.0 (18–44 years), 11.0 (45–69 years), and 10.0 (≥70 years). PCI scores ranged from 0 to 39, with no significant trend across continuous age (*p* = 0.407).

Complete subdiaphragmatic peritonectomies (right and/or left) were tracked as separate variables since the procedure can lead to a significant pleural effusion that can lead to respiratory complications and often requires pleural catheter/tube placement. Complete subdiaphragmatic right upper quadrant (RUQ) peritonectomy was performed in 31% of patients, with no significant difference between age groups (*p* = 0.218) or continuous age (*p* = 0.116). Similarly, left upper quadrant (LUQ) peritonectomy occurred in 9.6% of cases, with no significant association with age groups (*p* = 0.727) or continuous age (*p* = 0.509). These findings indicate that age did not influence the likelihood of upper quadrant peritonectomy, which is important as such procedures can lead to complications like reactive pleural effusions.

Bowel resections were performed in 76.4% of patients, including both low anterior resections (LARs) and other bowel resections. Specifically, 51% of patients had bowel resections other than LAR (*n* = 139), while LAR was documented in 25% of the cohort (*n* = 68). There was no significant difference in bowel resections by age group (*p* = 0.831) or continuous age (*p* = 0.744). Similarly, no significant association was found between LAR and age (*p* = 0.871, continuous *p* = 0.950).

The distribution of PCI volume categories across the cohort revealed that 48% of patients had low-volume PCI, 35% had moderate-volume PCI, and 17% had high-volume PCI (Table 1). No significant association was found between PCI volume and age groups (*p* = 0.627) or continuous age (*p* = 0.918).

**Table 1:** Patient characteristics in HIPEC procedures stratified by age groups: This table provides a summary of key surgical and demographic characteristics of patients undergoing HIPEC, stratified by age groups.

Characteristic	Overall, *n* = 271 ^1^	Age 18–44, *n* = 45 ^1^	Age 45–69, *n* = 190 ^1^	Age ≥70, *n* = 36 ^1^	*p*-Value (Categorical Age) ^2^	*p*-Value (Continuous Age) ^3^
Peritoneal Cancer Index (PCI)					0.544	0.407
Mean (SD)	11.3 (7.9)	10.0 (7.2)	11.6 (8.1)	11.6 (7.7)		
Median (IQR)	10.0 (5.0, 16.0)	9.0 (5.8, 14.0)	11.0 (4.0, 17.0)	10.0 (6.0, 15.0)		
Range	0.0, 39.0	0.0, 33.0	0.0, 39.0	2.0, 30.0		
Unknown	8	1	6	1		
Complete RUQ Peritonectomy					0.218	0.116
Yes	84 (31%)	9 (20%)	63 (33%)	12 (33%)		
Complete LUQ Peritonectomy					0.727	0.509
Yes	26 (9.6%)	4 (8.9%)	20 (11%)	2 (5.6%)		
Bowel Resection Performed (other than LAR)					0.831	0.744
Yes	139 (51%)	22 (49%)	97 (51%)	20 (56%)		
LAR Performed					0.871	0.950
Yes	68 (25%)	12 (27%)	46 (24%)	10 (28%)		
PCI Volume					0.627	0.918
Low Volume	126 (48%)	24 (55%)	85 (46%)	17 (49%)		
Moderate Volume	92 (35%)	16 (36%)		64 (35%)	12 (34%)	
High Volume	45 (17%)	4 (9.1%)	35 (19%)	6 (17%)		
Unknown	8	1	6	1		

^1^ *n* (%); ^2^ Pearson’s Chi-squared test; Fisher’s exact test; Kruskal–Wallis rank sum test; ^3^ linear regression; Kruskal–Wallis rank sum test.

### 3.3. Length of Stay (LoS)

The average length of stay (LoS) for patients undergoing CRS-HIPEC was 10.0 days (SD 8.7), with a median of 8.0 days (IQR: 7.0, 10.0) (Table 2). Patients aged 18–44 had a shorter mean LoS of 8.1 days (SD 3.3), compared to 10.2 days (SD 9.7) for those aged 45–69, and 10.9 days (SD 7.5) for patients aged ≥70. This difference was statistically significant across categorical age groups (*p* = 0.009), although it was not significant when evaluated as a continuous variable (*p* = 0.111) (Table 2).

**Table 2:** Clinical outcomes and survival metrics stratified by age groups for CRS-HIPEC patients.

Outcome	Overall, *n* = 271 ^1^	Age 18–44, *n* = 45 ^1^	Age 45–69, *n* = 190 ^1^	Age ≥70, *n* = 36 ^1^	*p*-Value (Categorical Age) ^2^	*p*-Value (Continuous Age) ^3^
Length of stay (LoS)					0.009	0.111
Mean (SD)	10.0 (8.7)	8.1 (3.3)	10.2 (9.7)	10.9 (7.5)		
Median (IQR)	8.0 (7.0, 10.0)	7.0 (6.0, 8.0)	8.0 (7.0, 10.0)	8.5 (7.0, 11.5)		
Range	3.0, 107.0	4.0, 17.0	3.0, 107.0	3.0, 47.0		
Discharge disposition					0.001	0.001
Home with and without assist	245 (90%)	45 (100%)	173 (91%)	27 (75%)		
Hospice	2 (0.7%)	0 (0%)	2 (1.1%)	0 (0%)		
Transitional institution	24 (8.9%)	0 (0%)	15 (7.9%)	9 (25%)		
Recurrence					0.878	0.764
No	128 (47%)	21 (47%)	89 (47%)	18 (51%)		
Yes	142 (53%)	24 (53%)	101 (53%)	17 (49%)		
Unknown	1	0	0	1		
If “yes” was there peritoneal recurrence?					0.716	0.566
No	153 (57%)	28 (62%)	106 (56%)	19 (54%)		
Yes	116 (82%)	17 (71%)	83 (83%)	16 (94%)		
Unknown	2	0	1	1		
Disease-free survival time (in days)					0.467	0.589
Mean (SD)	315.2 (253.9)	274.1 (250.5)	326.5 (260.3)	305.4 (225.1)		
Median (IQR)	237.0 (123.5, 411.0)	202.5 (102.5, 335.0)	252.5 (125.2, 418.5)	254.0 (181.0, 342.0)		
Range	42.0, 1365.0	52.0, 1126.0	42.0, 1365.0	82.0, 1029.0		
Unknown	128	21	88	19		
Peritoneal disease-free survival time (in days)					0.199	0.556
Mean (SD)	389.6 (332.0)	349.5 (431.0)	397.0 (308.0)	401.9 (330.9)		
Median (IQR)	302.0 (158.5, 493.0)	208.0 (102.5, 383.0)	350.0 (162.0, 497.5)	271.0 (210.0, 393.0)		
Range	42.0, 1996.0	52.0, 1996.0	42.0, 1813.0	82.0, 1096.0		
Unknown	128	21	88	19		
Mortality status					0.254	0.309
Alive	200 (74%)	36 (80%)	141 (74%)	23 (64%)		
Dead	71 (26%)	9 (20%)	49 (26%)	13 (36%)		
Survival length (in years)					0.824	0.665
Mean (SD)	1.8 (1.3)	1.6 (0.8)	1.9 (1.4)	1.7 (1.2)		
Median (IQR)	1.5 (0.9, 2.6)	1.8 (1.3, 4.4)	1.8 (0.8, 2.7)	1.4 (0.9, 2.1)		
Range	0.1, 5.9	0.6, 6.4	0.1, 5.9	0.4, 4.8		
Unknown	200	8	141	23		
Follow-up length alive (in years)					0.862	0.632
Mean (SD)	2.6 (1.6)	2.8 (1.8)	2.6 (1.6)	2.7 (1.6)		
Median (IQR)	2.3 (1.3, 3.8)	1.9 (1.3, 4.4)	2.9 (1.3, 3.7)	2.9 (1.3, 4.0)		
Range	0.1, 6.6	0.6, 6.4	0.1, 6.6	0.6, 5.8		
Unknown	61	8	42	11		
LoS categorical					0.012	0.060
<9 days	167 (62%)	36 (80%)	113 (59%)	18 (50%)		
9–13 days	75 (28%)	4 (8.9%)	58 (31%)	13 (36%)		
≥14 days	29 (11%)	5 (11%)	19 (10%)	5 (14%)		
Clavien–Dindo					0.151	0.008
C-D grade <3 **	226 (83%)	41 (91%)	158 (83%)	27 (75%)		
C-D grade ≥3	45 (17%)	4 (8.9%)	32 (17%)	9 (25%)		
Clavien–Dindo if multiple					0.185	0.015
0	226 (83%)	41 (91%)	158 (83%)	27 (75%)		
1	33 (12%)	2 (4.4%)	23 (12%)	8 (22%)		
>1	12 (4.4%)	2 (4.4%)	9 (4.7%)	1 (2.8%)		
Clavien–Dindo continuous					0.191	0.097
Mean (SD)	0.2 (0.6)	0.1 (0.5)	0.2 (0.7)	0.3 (0.5)		
Median (IQR)	0.0 (0.0, 0.0)	0.0 (0.0, 0.0)	0.0 (0.0, 0.0)	0.0 (0.0, 0.3)		
Range	0.0, 5.0	0.0, 2.0	0.0, 5.0	0.0, 2.0		
Readmission					0.041	0.006
No	204 (75%)	35 (78%)	148 (78%)	21 (58%)		
Yes	67 (25%)	10 (22%)	42 (22%)	15 (42%)		

^1^ *n* (%); ^2^ Kruskal–Wallis rank sum test; Fisher’s exact test; Pearson’s Chi-squared test; ^3^ linear regression; Kruskal–Wallis rank sum test; ** Of note Patients in the group with C-D grade <3 also included those with no complications (C-D grade 1, 2, or none).

### 3.4. Clavien–Dindo: Postoperative Complications

Clavien–Dindo complication grades ≥3 were reported in 17% of the cohort, with the highest incidence in patients aged ≥70 years (25%) (Table 2). Although the association with categorical age was not statistically significant (*p* = 0.151), continuous age analysis revealed a significant association (*p* = 0.008). These findings were further validated using logistic regression models (Table 3), which confirmed that patients with high PCI volumes were at significantly increased risk of complications (OR: 5.94, 95% CI: 1.87–19.9, *p* = 0.003).

**Table 3:** Risk factors for severe complications (Clavien–Dindo grade ≥3) in HIPEC patients: logistic regression model.

Characteristic	OR ^1^	95% CI ^1^	*p*-Value
Age Categorical			
Age 18–44	-	-	
Age 45–69	1.64	0.48, 7.64	0.468
Age ≥70	2.50	0.49, 14.7	0.278
Sex			
Female	-	-	
Male	1.04	0.42, 2.52	0.935
PCI Volume			
Low Volume	-	-	
Moderate Volume	2.29	0.82, 6.69	0.118
High Volume	5.94	1.87, 19.9	0.003
Colorectal Tumor			
LAMN	-	-	
Other Colorectal Tumor	2.96	0.90, 11.1	0.085
R Colon Tumor	3.88	1.20, 14.4	0.030
Rectal	9.42	0.91, 89.7	0.047

^1^ OR = Odds Ratio, CI = Confidence Interval.

### 3.5. Discharge Disposition

Discharge disposition varied significantly by age group. Patients aged 18–44 years were more likely to be discharged home with or without assistance (100%), while patients aged ≥70 years had higher rates of discharge to transitional institutions (25%) (Table 2). This difference was statistically significant for both categorical (*p* = 0.001) and continuous age (*p* = 0.001).

### 3.6. Disease-Free Survival and Recurrence

The mean disease-free survival (DFS) was 315.2 days (SD 253.9) across the cohort, with the longest DFS in patients aged 45–69 years (326.5 days, SD 260.3). However, DFS did not differ significantly between age groups (*p* = 0.467) or as a continuous variable (*p* = 0.589) (Table 2).

Recurrence was observed in 53% of patients, with no significant difference by age group (*p* = 0.878) or continuous age (*p* = 0.764) (Table 2). Multivariate analysis (Table 4) showed that both moderate and high PCI volumes were independently associated with increased recurrence risk. Moderate PCI volume had a hazard ratio (HR) of 2.28 (95% CI: 1.37–3.77, *p* = 0.001), while high PCI volume had an HR of 3.80 (95% CI: 2.04–7.09, *p* < 0.001). Among colorectal tumors, right colon tumors exhibited the highest recurrence risk (HR: 7.18, 95% CI: 3.70–14.0, *p* < 0.001).

**Table 4:** Risk factors for recurrence in HIPEC patients: multivariate analysis.

Characteristic	HR ^1^	95% CI ^1^	*p*-Value
Age Categorical			
Age 18–44	-	-	
Age 45–69	0.63	0.35, 1.12	0.115
Age ≥70	0.96	0.43, 2.13	0.913
Sex			
Female	-	-	
Male	0.89	0.57, 1.40	0.616
PCI Volume			
Low Volume	-	-	
Moderate Volume	2.28	1.37, 3.77	0.001
High Volume	3.80	2.04, 7.09	<0.001
Colorectal Tumor			
LAMN	-	-	
Other Colorectal Tumor	5.57	2.87, 10.8	<0.001
R Colon Tumor	7.18	3.70, 14.0	<0.001
Rectal	1.69	0.22, 13.1	0.616

^1^ HR = Hazard Ratio, CI = Confidence Interval.

### 3.7. Peritoneal Recurrence

Peritoneal recurrence was observed in 82% of cases with recurrence, with no statistically significant differences across categorical age groups (*p* = 0.716) or as a continuous variable (*p* = 0.566) (Table 2). However, Cox proportional hazard models (Table 5) indicated that both moderate and high Peritoneal Cancer Index (PCI) volumes were significantly associated with an increased risk of peritoneal recurrence. Patients with moderate PCI volume had a hazard ratio of 3.42 (95% CI: 1.82–6.64, *p* < 0.001), while those with high PCI volume had a hazard ratio of 6.67 (95% CI: 3.27–13.6, *p* < 0.001), compared to patients with low PCI volume. Additionally, recurrence risk varied significantly across primary tumor types, with right colon tumors (HR: 7.55, 95% CI: 3.53–16.2, *p* < 0.001) and other colorectal tumors (HR: 6.82, 95% CI: 3.17–14.7, *p* < 0.001) showing a markedly higher risk of peritoneal recurrence compared to low-grade appendiceal mucinous neoplasms (LAMNs).

**Table 5:** Risk factors for peritoneal recurrence in HIPEC patients. Logistic regression model ^†^.

Characteristic	OR ^1^	95% CI ^1^	*p*-Value
Age Categorical			
Age 18–44	-	-	
Age 45–69	0.71	0.34, 1.49	0.370
Age ≥70	1.40	0.55, 3.59	0.483
Sex			
Female	-	-	
Male	0.79	0.47, 1.35	0.394
PCI Volume			
Low Volume	-	-	
Moderate Volume	3.42	1.82, 6.64	<0.001
High Volume	6.67	3.27, 13.6	<0.001
Colorectal Tumor			
LAMN	-	-	
Other Colorectal Tumor	6.82	3.17, 14.7	<0.001
R Colon Tumor	7.55	3.53, 16.2	<0.001
Rectal	2.42	0.30, 19.7	0.408

^1^ OR = Odds Ratio, CI = Confidence Interval. † Data presented in this table includes only patients with colorectal and low-grade appendiceal mucinous neoplasm (LAMN) tumors.

Peritoneal disease-free survival (PDFS) time also varied across the cohort. The mean PDFS was 389.6 days (SD 332.0), with no statistically significant differences across age groups (*p* = 0.199) or continuous age (*p* = 0.556). The median PDFS was 302.0 days (IQR: 158.5, 493.0), with the shortest PDFS observed in younger patients (208.0 days, IQR: 102.5, 383.0) and the longest in patients aged 45–69 (350.0 days, IQR: 162.0, 497.5).

### 3.8. Mortality and Readmission Rates

Mortality rates did not differ significantly by age group (*p* = 0.254 for categorical age, *p* = 0.309 for continuous age) (Table 2). As of the September 2024 review, 26% of the cohort was deceased. The survival length, defined as the time from surgery to death, averaged 1.8 years (SD 1.3) across the cohort, with no significant differences by age (*p* = 0.824 for categorical age, *p* = 0.665 for continuous age). The median survival length was 1.5 years (IQR: 0.9–2.6). The multivariate survival analysis revealed no significant differences in mortality rates among HIPEC patients when categorized by age, sex, or PCI volume. Although patients aged 45–69 years exhibited a slightly elevated hazard ratio (HR: 1.30; 95% CI: 0.79–2.14) compared to younger patients, this was not statistically significant (*p* = 0.299) (Table 6). Similarly, male patients and those with moderate or high PCI volumes did not demonstrate significantly higher hazard ratios compared to their counterparts. Regarding tumor characteristics, right colon and rectal tumors exhibited slightly elevated hazard ratios (HR: 1.20 and 1.27, respectively), but these findings were also not statistically significant (Table 6).

There were no in-hospital deaths observed among patients in this cohort.

**Table 6:** Multivariate survival analysis of HIPEC patients by demographic and clinical factors.

Characteristic	HR ^1^	95% CI ^1^	*p*-Value
Age Categorical			
Age 18–44	-	-	
Age 45–69	1.30	0.79, 2.14	0.299
Age ≥70	0.96	0.47, 1.95	0.912
Sex			
Female	-	-	
Male	1.16	0.79, 1.70	0.451
PCI Volume			
Low Volume	-	-	
Moderate Volume	1.06	0.69, 1.62	0.796
High Volume	1.18	0.71, 1.99	0.522
Colorectal Tumor			
LAMN	-	-	
Other Colorectal Tumor	0.92	0.59, 1.43	0.718
R Colon Tumor	1.20	0.75, 1.93	0.438
Rectal	1.27	0.44, 3.72	0.657

^1^ HR = Hazard Ratio, CI = Confidence Interval.

Follow-up length alive refers to the duration from the date of surgery to the last recorded visit for patients who remain alive. On average, this period was 2.6 years (with a standard deviation of 1.6 years), and statistical analysis showed no significant differences in follow-up duration based on age, whether assessed by age categories (*p* = 0.862) or as a continuous variable (*p* = 0.632), as indicated in Table 2. The median follow-up length alive was calculated at 2.3 years, with an interquartile range (IQR) spanning from 1.3 to 3.8 years.

Readmissions occurred in 25% of patients, with significantly higher rates among patients aged ≥70 years (42%) compared to younger patients (22%) (*p* = 0.041 for categorical age, *p* = 0.006 for continuous age) (Table 2).

### 3.9. Clinical Outcomes and Survival Metrics by Tumor Location

Table 7 highlights clinical outcomes and survival metrics stratified by tumor location into three groups: colorectal and appendiceal tumors (Group 1 or CA group), gastrointestinal and small organ tumors (Group 2 or GSO Group), and gynecologic and mesothelial tumors (Group 3 or GM Group).

The age distribution was similar across groups, with a mean age of 56.7 years (SD 12.0) and a median of 58 years (IQR: 49.0–65.0). Patients aged 45–69 years comprised the largest proportion in all groups (70% overall), while patients aged ≥70 years accounted for 13%. Age differences across groups were not statistically significant (*p* = 0.940).

The mean length of stay (LOS) differed across the groups, with GSO Group patients experiencing the longest mean LOS at 14.2 days (SD 13.4), compared to 10.1 days (SD 9.1) for CA Group and 8.1 days (SD 3.5) for GM Group (*p* = 0.006). Recurrence rates were highest in GSO Group (75%), followed by GM Group (57%) and CA Group (50%), though these differences were not statistically significant (*p* = 0.189). Disease-free survival (DFS) varied across the groups, with the shortest median DFS observed in GSO Group (212 days, IQR: 92.0–252.0) compared to CA Group (222 days, IQR: 120.5–407.5) and GM Group (288 days, IQR: 172.5–481.0), though the differences were not significant (*p* = 0.451).

Of those who experienced recurrence, peritoneal recurrence occurred in 67% of GSO Group patients, 51% of GM Group patients, and 40% of CA Group patients (*p* = 0.093). Mortality rates were similar across the groups, with 25% of patients deceased in GSO Group, 30% in GM Group, and 25% in CA Group (*p* = 0.859). The length of survival after surgery did not vary significantly, with mean survival times of 1.9 years (SD 1.4) in CA Group, 1.4 years (SD 0.6) in GSO Group, and 1.7 years (SD 1.1) in GM Group 3 (*p* = 0.890).

Discharge disposition also showed variability. Most patients were discharged home with or without assistance: 90% in CA Group, 83% in GSO Group, and 94% in GM Group. Discharge to transitional institutions was more frequent in GSO Group (17%) compared to CA Group (9.0%) and GM Group (6.4%) (*p* = 0.653).

**Table 7:** Clinical outcomes and survival metrics stratified by tumor location in CRS-HIPEC patients ^†^.

Characteristic	Overall, *n* = 271 ^1^	Group 1, *n* = 212 ^1^	Group 2, *n* = 12 ^1^	Group 3, *n* = 47 ^1^	*p*-Value ^2^
Age					0.940
Mean (SD)	56.7 (12.0)	56.8 (12.2)	55.8 (12.0)	56.8 (11.1)	
Median (IQR)	58.0 (49.0, 65.0)	58.0 (49.0, 65.2)	60.0 (49.0, 63.2)	58.0 (50.0, 65.0)	
Range	18.0, 82.0	18.0, 82.0	31.0, 72.0	29.0, 78.0	
LoS					0.006
Mean (SD)	10.0 (8.7)	10.1 (9.1)	14.2 (13.4)	8.1 (3.5)	
Median (IQR)	8.0 (7.0, 10.0)	8.0 (7.0, 10.0)	9.5 (8.0, 12.5)	7.0 (6.0, 9.5)	
Range	3.0, 107.0	4.0, 107.0	7.0, 55.0	3.0, 22.0	
Discharge disposition					0.653
Home with and without assist	245 (90%)	191 (90%)	10 (83%)	44 (94%)	
Hospice	2 (0.7%)	2 (0.9%)	0 (0%)	0 (0%)	
Transitional institution	24 (8.9%)	19 (9.0%)	2 (17%)	3 (6.4%)	
Recurrence					0.189
Yes	142 (53%)	106 (50%)	9 (75%)	27 (57%)	
Unknown	1	1	0	0	
Disease-free survival time (in days)					0.451
Mean (SD)	315.2 (253.9)	314.9 (263.9)	237.6 (164.1)	342.1 (238.8)	
Median (IQR)	237.0 (123.5, 411.0)	222.0 (120.5, 407.5)	212.0 (92.0, 252.0)	288.0 (172.5, 481.0)	
Range	42.0, 1365.0	51.0, 1365.0	76.0, 513.0	42.0, 1029.0	
Unknown	128	105	3	20	
If there was recurrence was there Peritoneal recurrence?					0.093
Yes	116 (43%)	84 (40%)	8 (67%)	24 (51%)	
Unknown	2	2	0	0	
Mortality status					0.859
Alive	200 (74%)	158 (75%)	9 (75%)	33 (70%)	
Dead	71 (26%)	54 (25%)	3 (25%)	14 (30%)	
Age categorical					0.718
Age 18–44	45 (17%)	37 (17%)	3 (25%)	5 (11%)	
Age 45–69	190 (70%)	146 (69%)	8 (67%)	36 (77%)	
Age ≥70	36 (13%)	29 (14%)	1 (8.3%)	6 (13%)	
LoS categorical					0.085
<9 days	167 (62%)	130 (61%)	5 (42%)	32 (68%)	
9–13 days	75 (28%)	57 (27%)	4 (33%)	14 (30%)	
≥14 days	29 (11%)	25 (12%)	3 (25%)	1 (2.1%)	
Clavien–Dindo					>0.999
C-D grade <3	226 (83%)	177 (83%)	10 (83%)	39 (83%)	
C-D grade ≥3	45 (17%)	35 (17%)	2 (17%)	8 (17%)	
Clavien–Dindo if multiple					0.749
0	226 (83%)	177 (83%)	10 (83%)	39 (83%)	
1	33 (12%)	25 (12%)	1 (8.3%)	7 (15%)	
>1	12 (4.4%)	10 (4.7%)	1 (8.3%)	1 (2.1%)	
Clavien–Dindo continuous					0.995
Mean (SD)	0.2 (0.6)	0.2 (0.6)	0.5 (1.4)	0.2 (0.4)	
Median (IQR)	0.0 (0.0, 0.0)	0.0 (0.0, 0.0)	0.0 (0.0, 0.0)	0.0 (0.0, 0.0)	
Range	0.0, 5.0	0.0, 4.0	0.0, 5.0	0.0, 2.0	
Readmission					0.399
Yes	67 (25%)	56 (26%)	3 (25%)	8 (17%)	
Peritoneal disease-free survival Time (in days)					0.406
Mean (SD)	389.6 (332.0)	401.3 (356.3)	253.6 (173.8)	388.8 (261.1)	
Median (IQR)	302.0 (158.5, 493.0)	309.0 (153.5, 497.0)	212.0 (92.0, 382.0)	347.0 (188.5, 501.5)	
Range	42.0, 1996.0	51.0, 1996.0	76.0, 527.0	42.0, 1029.0	
Unknown	128	105	3	20	
Survival length					0.890
Mean (SD)	1.8 (1.3)	1.9 (1.4)	1.4 (0.6)	1.7 (1.1)	
Median (IQR)	1.5 (0.9, 2.6)	1.7 (0.9, 2.6)	1.5 (1.2, 1.8)	1.6 (0.9, 2.5)	
Range	0.1, 5.9	0.1, 5.9	0.8, 2.0	0.1, 3.3	
Unknown	200	158	9	33	
Follow-up length alive					0.224
Mean (SD)	2.6 (1.6)	2.7 (1.6)	2.1 (1.7)	2.3 (1.3)	
Median (IQR)	2.3 (1.3, 3.8)	2.5 (1.3, 3.8)	1.8 (0.8, 2.4)	1.9 (1.2, 3.6)	
Range	0.1, 6.6	0.1, 6.6	0.5, 5.5	0.7, 4.9	
Unknown	61	46	2	13	

^1^ *n* (%) ^2^ Kruskal–Wallis rank sum test; Fisher’s exact test; Pearson’s Chi-squared test. † Clinical outcomes and survival metrics are stratified into three groups: colorectal and appendiceal tumors (Group 1 or CA Group), gastrointestinal and small organ tumors (Group 2 or GSO Group), and gynecologic and mesothelial tumors (Group 3 or GM Group).

## 4. Discussion

Age has historically been regarded as a limiting factor for aggressive abdominal surgical therapies, including CRS-HIPEC, which is utilized in managing peritoneal surface malignancies. However, recent studies challenge this view, showing that patients across all age groups, including elderly populations, can achieve favorable outcomes [10,11,12]. Wong et al. reported no significant increase in high-grade complications between elderly and non-elderly patients undergoing CRS-HIPEC (44.4% vs. 24.5%, *p* = 0.79), suggesting that age alone should not contraindicate the procedure [13]. Similarly, Beckert et al. found that while older patients experienced more overall complications (76.0% vs. 47.0%, *p* = 0.048), there was no significant increase in severe complications (21.0% vs. 12.0%, *p* = 1.000) [14].

This study is consistent with previous research, showing that age did not significantly impact mortality, morbidity, or recurrence. However, length of stay, readmission rates, and discharge disposition were significantly different across age groups (Table 2). Older patients had longer hospital stays, higher readmission rates, and were more frequently discharged to transitional care facilities. These findings suggest that while age alone may not predict long-term oncological outcomes in CRS-HIPEC patients, enhanced postoperative care is essential for older patients.

To further optimize perioperative management for elderly patients, several strategies should be emphasized. Preoperative assessments should include a comprehensive evaluation of comorbidities, nutritional status, and functional capacity to identify and address potential risk factors proactively. Enhanced Recovery After Surgery (ERAS) protocols tailored to elderly patients can help minimize surgical stress and expedite recovery. Postoperative care should involve close monitoring for complications, early mobilization, and coordination with physical therapy and nutrition services to support recovery. Lastly, structured follow-up programs, tailored discharge plans, and coordinated communication with transitional care facilities may help reduce readmissions and improve recovery outcomes for this demographic. These strategies not only improve outcomes but also mitigate the risks of prolonged hospitalization and readmissions for elderly CRS-HIPEC patients.

Tumor location influenced clinical outcomes and survival metrics. Patients with gastrointestinal and small organ tumors (Group 2) experienced the longest hospital stays, highest recurrence rates, and shortest disease-free survival times compared to those with colorectal and appendiceal tumors (Group 1) or gynecologic and mesothelial tumors (Group 3). Additionally, Group 2 had the highest proportion of peritoneal recurrence, suggesting a more aggressive disease course that may require intensified surveillance and individualized management strategies. These findings underscore the complex relationship between tumor biology and surgical outcomes, advocating for tumor-specific considerations in perioperative planning and follow-up care.

The logistic regression models provide further insight into postoperative complications and recurrence. There was no significant association between age and C-D complications, although older patients showed a non-significant trend toward higher-grade complications (Table 3). This suggests that factors other than age, such as comorbidities and PCI volume, may contribute to postoperative outcomes. PCI scores were evenly distributed across age groups, indicating that the extent of peritoneal disease did not vary significantly with age (Table 1). These findings reinforce the feasibility of CRS-HIPEC across all age ranges, provided patients receive appropriate perioperative care. In addition to morbidity and mortality, these findings need to be considered in patient selection and should guide honest discussions related to life-bearing outcomes in older patients.

CRS-HIPEC demonstrates varying efficacy across different types of cancer, highlighting the importance of tailoring treatment based on tumor origin [15,16,17]. Previous studies have shown favorable outcomes for certain cancers, such as colorectal and ovarian cancers, with median survival times of 32 to 63 months and 45.7 months, respectively [18,19]. However, survival outcomes can vary significantly across different primary tumor sites, reinforcing the need to evaluate each patient’s case individually when considering CRS-HIPEC [20]. In this study, survival and recurrence analyses did not explore each primary tumor location separately, though the findings align with the existing literature suggesting that certain tumor types, such as mesothelioma and pseudomyxoma peritonei, benefit significantly from complete cytoreduction. Five-year survival rates exceeding 50% and 60–90% for these cancers underscore the potential of CRS-HIPEC when cytoreduction is thorough [21]. This study further supports the concept of patient selection for CRS-HIPEC based on cancer type and extent of disease, advocating for tailored approaches in clinical decision-making.

The Clavien–Dindo classification system offers a standardized approach to evaluating complications, facilitating comparisons across studies [22]. While categorical age was not significantly associated with C-D scores, continuous age analysis revealed a significant association. These findings emphasize the importance of individualized care pathways for patients undergoing CRS-HIPEC to manage potential complications effectively.

The primary oncological objective of CRS-HIPEC is to reduce peritoneal surface recurrence. Our analysis demonstrates that age does not independently influence recurrence, survival, or complication outcomes (Table 3, Table 4, Table 5, Table 6 and Table 7). Instead, primary organ involvement and PCI volume significantly impact patient outcomes. As shown in Table 4, colorectal tumors were associated with a higher risk of recurrence compared to LAMNs, with right colon tumors showing the greatest hazard risk and other colorectal tumors (transverse, left, and sigmoid colon) also exhibiting elevated recurrence risk. Additionally, rectal tumors had higher odds of severe complications as noted in Table 3. These findings highlight the importance of tumor location and disease burden in determining recurrence and complication outcomes following CRS-HIPEC.

Lastly, peritoneal recurrence rates were significantly influenced by PCI volume. Table 5 demonstrates that both moderate and high PCI volumes were associated with a higher risk of peritoneal disease recurrence. Table 3 also shows that high PCI volume was associated with an increased risk of severe complications.

Overall, this study reveals that while age does not independently impact critical outcomes in CRS-HIPEC patients, it is associated with higher readmission rates, longer hospital stays, and increased reliance on transitional care in older patients—factors that significantly affect both life expectancy and quality of life. Furthermore, our findings highlight the importance of primary tumor location and PCI volume as critical factors influencing recurrence and complications.

### Study Limitations

This study has limitations that merit consideration. The retrospective design introduces selection bias, favoring patients with better baseline health and fewer comorbidities, particularly among older patients. While this may influence clinical outcomes and limit generalizability, it highlights the importance of developing standardized surgical indications for CRS-HIPEC in future research.

The unequal distribution of patients among age groups, with a larger proportion aged 45–69 years, may reduce statistical power to detect differences between groups. Studies with more balanced age distributions are needed to validate these findings. Preoperative nutritional status, a key determinant of postoperative outcomes, was not systematically assessed. Although our institution implements nutritional optimization strategies, standardized metrics would enable a deeper understanding of their impact on CRS-HIPEC outcomes.

Notably, the single-center design offers the advantage of controlling for surgeon- and institution-related variabilities, as all operations were performed by the same surgeon, and patient recovery occurred within a consistent institutional framework. This consistency enhances the reliability of our findings within the study’s context.

Despite these limitations, this study provides valuable insights into the nuanced relationship between age and surgical outcomes in CRS-HIPEC for peritoneal carcinomas. Future multi-institutional studies are warranted to confirm these findings and explore strategies to optimize perioperative management, particularly for elderly patients. The data presented here lay the groundwork for generating hypotheses and designing prospective randomized controlled trials (RCTs) to refine patient selection, surgical techniques, and perioperative care in CRS-HIPEC patients.

## 5. Conclusions: A Look into the Future

The findings from this study highlight that a comprehensive assessment of PCI volume, tumor location, and individual patient needs is essential in CRS-HIPEC decision-making. Rather than relying on age-based criteria, clinicians should focus on personalized care plans, particularly for older patients who may benefit from structured rehabilitation and transitional care coordination to reduce re-admissions and enhance recovery. Future CRS-HIPEC clinical studies need to measure and include quality of life as an important outcome utilizing currently available tools [23]. When considering oncologic outcomes such as disease-free and overall survival, future research should delve into the biology of primary tumors and their cellular origin, as understanding the molecular and cellular characteristics of the tumor could help refine patient selection and optimize treatment efficacy. Tailoring CRS-HIPEC based on tumor biology and primary cancer cell location may ultimately improve outcomes and lead to more precise therapeutic approaches for peritoneal malignancies.

## Data Availability

The data presented in this study are available on request from the corresponding author due to the privacy of patients and the medical center.

## Abbreviations

The following abbreviations are used in this manuscript:

HIPEC	Hyperthermic Intraperitoneal Chemotherapy
CRS	Cytoreductive Surgery
PCI	Peritoneal Cancer Index
CC	Completeness of Cytoreduction
C-D	Clavien–Dindo Classification
DFS	Disease-Free Survival
PDFS	Peritoneal Disease-Free Survival
LAMN	Low-Grade Appendiceal Mucinous Neoplasm

## Appendix A. Peritoneal Cancer Index [24]

	**Regions**			
	0	Central		
	1	Right Upper		
	2	Epigastrium		
	3	Left Upper		
	4	Left Flank		
	5	Left Lower		
	6	Pelvis		
	7	Right Lower		
	8	Right Flank		
	9	Upper Jejunum		
	10	Lower Jejunum		
	11	Upper Ileum		
	12	Lower Ileum		
	
			**Lesion Size Score**	
			0	No Tumor
			1	Tumors up to 0.5 cm
			2	Tumors up to 5 cm
			3	Tumors ≥5 cm or confluence

## Appendix C. The Clavien–Dindo Classification System [26]

**Grade**	**Definition**
Grade I	Any deviation from the normal postoperative course not requiring surgical, endoscopic, or radiological intervention. This includes the need for certain drugs (e.g., antiemetics, antipyretics, analgesics, diuretics, and electrolytes), treatment with physiotherapy, and wound infections that are opened at the bedside.
Grade II	Complications requiring drug treatments other than those allowed for Grade I complications. This includes blood transfusion and total parenteral nutrition.
Grade III	Complications requiring surgical, endoscopic, or radiological intervention.
IIIa	Intervention not under general anesthetic.
IIIb	Intervention under general anesthetic.
Grade IV	Life-threatening complications (including CNS complications (e.g., brain hemorrhage, ischemic stroke, subarachnoid hemorrhage) that require intensive care or intensive care unit management but excludes transient ischemic attacks (TIAs).
IVa	Single-organ dysfunction (including dialysis).
IVb	Multi-organ dysfunction.
Grade V	Death of a patient.
